# Chloride of Sodium in the Ulcerative Stages of Typhoid Fever

**Published:** 1867-04

**Authors:** Luther C. White

**Affiliations:** Van Buren, Ark.


### Correspondence.

VAN BUREN, ARK., *Feb. 12, 1867.*

PROF. DAVIS—*My Dear Sir:*

After the reïteration of expressions of obligation to you personally, and my profound regard for your eminent services to the profession and to the public, permit me to relate to you my observations in the use of *chloride of sodium* in the ulcerative stages of typhoid fever.

During the summer and early autumn of 1861, this community was visited with one of those epidemics of remittent and typhoid fevers, or rather with that pernicious form of bilious remittent fever, which speedily runs into a typhoid form, with extensive ulceration of the glands of the bowels. My first case was that of a young gentleman, a merchant of this city, Mr. M. Hinkle, aged 22, attacked Aug. 1st, with the ordinary chill, followed by continued fever and immense discharges per anum, a few days thereafter, of blood and pus. I tried the routine treatment all to no purpose, so far as controlling the latter symptom, when it occurred to me to try COMMON SALT. Taking a saturated solution of the article, I gave two teaspoonfuls every 20 minutes, and continued about 3 hours, with a most decided and remarkable result. At the time of commencing its use, which was on the ninth day after the attack, and the sixth day after the supervention of the ulceration, my patient was exceedingly low, and the ulceration, judging from the quantity of blood and pus, was extensive. I continued the use of the remedy for fifteen days before I could finally dispense with it, and, to make assurance doubly sure, I omitted its use for several times, when the discharges would invariably return. I concluded that the common salt had saved my patient's life, and at this time I entertain not the slightest doubt about it.

So well pleased was I with the result that I subsequently used it in 17 cases, all with the like decided and beneficial results. There were no unpleasant symptoms—neither nausea, thirst, or anything else, save that it acted like a charm in every instance, and I saved every one of my patients.

Now, I do not know whether any other physician has tried the article in like cases or not, but I would strongly advise them to do so. Shut in as we have been, until recently, we could not tell what was going on outside in the moral world, nor what discoveries have been made in medicine ; but I do not recollect ever to have heard of common salt being used in fevers of any kind. I should be happy to know that other physicians have tested it. I often, in the year '61, gave it in advance of the ulceration, and in every case it controlled and prevented it. Indeed, I thought at the time that I had made a most valuable as well as wonderful discovery, for I used it in so many instances, and proved it, that I have no doubt whatever that it saved life where nothing else would have done it. It must not, of course, be understood that it was the sole remedy used in those cases, for I gave tonics, alteratives, opiates, terebentha, etc., etc.; but nothing would control the ulceration but the salt, and that did it to perfection.

Yours very truly, LUTHER C. WHITE, M.D.

